# Everybody's Page

**Published:** 1888-07-28

**Authors:** 


### Everybody's Page.

A plague which broke out at Naples in 1656 carried off 400,000 of the population in six months.

A South Florida paper predicts that as the poppy grows luxuriantly in that section, the production of opium will one day be one of the States' great industries.

A four-year-old American gave an admirable definition of influenza when he complained to his mother, "Bofe of my eyes is leaking, and one of my noses don't go."

During last year 993 bodies were cremated in Buenos Ayres, of which number 742 were those of persons who had died of cholera, small-pox, and other contagious diseases.

For the purpose of preserving milk for some time, so as to keep it in good condition for analysis, E. Schroder recommends the addition to it of one drop of essential oil of mustard, or more, according to the quantity of milk.

Fumigation is said to have originated with Acron, a physician of Agrigentum, who first caused great fires to be lighted and aromatics to be thrown into them to purify the air, and thus to have stopped the plague at Athens and other places in Greece about 473 B.C.

It is said that the deaf are not as a rule subject to attacks of sea-sickness, nor liable to become dizzy from swinging, or standing in narrow and high places; but, on the other hand, very few of them can walk with steady steps at night. A respectable deaf mute was followed to his home one night by a policeman, who thought that his unsteady gait was the result of intoxication—a most unfounded suspicion. No explanation has yet been found for this curious phenomenon.

A curious case is reported of a gentleman living in Atalanta, who has one eye dark blue in colour while the other is light-grey. In the daytime—from sunrise to sunset—he cannot see anything with the blue eye, but can see distinctly with the grey one, while between sunset and sunrise only the blue eye is of any use. His hearing is similarly affected. He can hear only with the blind side, so that he hears with one ear by day and another by night.

Dr. W. Faust praises the prompt effects of antifebrine as remedy for almost any kind of headache, provided it is taken in sufficient amount. He states that he finds it useless to discriminate between the various causes of headache, so far as this remedy is concerned, as it gives relief in nearly every case. Antifebrine is not, however, a thing for amateurs to meddle with incautiously. The *Industrie Blätter* quotes the case of a woman who, on the recommendation of an advertisement, took 60 grains of the drug in two doses, at short intervals. After three hours she lapsed into a deep coma, from which she had to be roused by strong stimulants.

Dr. Alice Sollier has just published a book dealing with defects of the teeth commonly found in idiots and feeble-minded children. It seems to be the rule that when nature leaves the mental faculties incomplete she signs her work with some physical defect. The teeth frequently bear this mark. Sometimes they are too small, often they are grooved, either perpendicularly or horizontally, and occasionally they are set in jaw bones of abnormal form. Dr. Sollier has observed in many cases apparent erosions, caused, however, not by the enamel of the teeth being eaten away by an acid, but by its never having been formed. In fact, all these deformities may be looked on as evidences of arrested development.

A Commission has recently been sitting in Paris under the auspices of the *Conseil d'Hygiene Publique* of the Seine department to inquire into the advisability of employing saccharine instead of sugar in articles of food. The conclusions to which the committee have come are, as stated in their report, that "the introduction of saccharine into alimentary substances should be forbidden as dangerous to public health. Saccharine may render services as a medicine, but it cannot be regarded as a food." The report further says, "Saccharine gives only the illusion of sugar." This is, in fact, at once its merit and its defect.

M. Casa Longa, editor of *La Chronique Industrielle*, seems to have an exaggerated horror of hydrophobia. In order to prevent it he would like to forbid the existence of dogs in towns, or at least to use the most stringent measures to prevent their ever being seen in the streets. We think his idea a little extreme, though we do not go so far towards the other side as a certain dog-loving friend of ours, who hearing of a case where a much-abused terrier had at last—even a worm will turn, much more a Dandie Dinmont—turned and bitten the small boy who had teased him—hearing of this, we say, our friend remarked: "As a matter of justice, the obvious way to prevent such accidents is to poison the child." But the child's parents poisoned the dog instead.

Surgeon-Major E. Lawrie, in his report on medical administration in the dominions of the Nizam during 1887, gives a curious instance of the strength of the prejudice against amputation of a limb which survices among semi-civilised people. A native was severely bitten in the foot by a tiger, and after an attempt to save the foot had been made the patient consented to amputation, but his friends objected that he would be no use without a foot, and that they would rather he should die. "As the patient was old enough," writes Dr. Lawrie, "to judge for himself, I turned the friends out of the hospital and took the foot off. They returned, armed to the teeth, after the operation was completed; and if it had not been for a very strong guard, would certainly have attacked us. Being foiled in this, they entirely deserted the man whose foot had been amputated. We were obliged to keep him in the hospital for months, as he could get no food outside; and if his Highness the Nizam had not taken him into his service, he would have been there still, or died of starvation."

A correspondent of the *Echo* has discovered how to bring up hospital incomes to hospital needs. The notion has all the sublime simplicity of genius. Let the hospitals of London unite—no one institution is to profit by this scheme to the exclusion of others—to purchase all the automatic "try your weight" machines that attract our gaze, and sometimes our coppers, at railway stations. It is an exquisite idea! You stand on the plate of the machine, drop your penny into the slot, according to instructions, and thereby enrich a number of impoverished and deserving institutions to the extent of a fraction of a penny, and gain an interesting piece of personal information, which has, occasionally, a distinct hygienic value. Could anything be more attractive? What a pity it is the idea was not hit upon before! Now that we have got it, however, let us carry it out to the full. Let us satisfy, not only the adipose curiosity of old Twentystun, but the sweet, and somewhat sticky, desires of youth, and the nicotian longings of manhood. There are the post-card machines too. They pay the best, because you have to give a penny for a thing the strict market value of which is only three farthings. It is true that the hospitals will have to go to an initial expense of £60,000 to buy up the machines; but what of that!

